# PCD Appoints New Experts and Releases Four Supplements in 2020 on COVID-19, CDC High Obesity Program, Public Health and Pharmacy, and Mental Health

**DOI:** 10.5888/pcd17.200559

**Published:** 2020-12-17

**Authors:** Leonard Jack

**Affiliations:** 1 Preventing Chronic Disease, Office of Medicine and Science, National Center for Chronic Disease Prevention and Health Promotion, Centers for Disease Control and Prevention, Atlanta, Georgia

This year has been an incredible one for Preventing Chronic Disease (*PCD*). The journal continues to publish timely peer-reviewed articles that serve as a vital resource to researchers, evaluators, practitioners, and policy makers. To assist us in continuing this mission, we have appointed an impressive group of volunteers who agreed to join *PCD’s* team of Associate Editors, our Editorial Board, and our Statistics Review Committee. These new appointees will help us advance our vision to disseminate proven and promising peer-reviewed public health findings, innovations, and practices with editorial content respected for its integrity and relevance to chronic disease prevention. Over the coming years, *PCD* looks forward to working closely with this diverse group of public health professionals, who bring an abundance of expertise across a wide range of research and practice areas. For more information on our Associate Editors, Editorial Board, and Statistics Review Committee members, please visit https://www.cdc.gov/pcd/about_the_journal/index.htm.

Despite the challenges associated with working during a pandemic, *PCD* published 4 important collections this year. In March we published a collection of articles on CDC’s High Obesity Program. From 2014 through 2018, that program provided funding to 11 land grant universities (LGUs) in states with counties in which the prevalence of adult obesity was greater than 40%, according to the 2013 Behavioral Risk Factor Surveillance System. CDC staff members with expertise in obesity provided technical assistance and guidance to those universities on evidence-based nutrition and physical activity interventions, community-based participatory approaches, community needs assessments, coalition development, and performance measures, and they leveraged resources. The 9 articles featured in this collection discuss public health strategies aimed at improving access to healthy food and opportunities for physical activity by working with nontraditional partners and by using community-based participatory approaches to engage communities (1).

In response to the pandemic, *PCD* released a special supplement in August, *US Public Health Response to COVID-19 and Chronic Disease: Continuing the Commitment to Improve Population Health *(2). The supplement offers 16 commentaries generated by individuals working on the front lines of the pandemic. These authors represent an impressive mix of expertise in public health, medicine, infectious disease, health disparities, health equity, community engagement, community organization, nursing, pharmacy, oral public health, health communication, health system change, environmental health, geographic information systems, geospatial analyses, and more. They share expertise on the bidirectional relationship between chronic disease and novel coronavirus disease 2019 (COVID-19), its impact on population health in the United States and around the world, and early thinking on emerging public health approaches to address COVID-19 and chronic disease.

Then in September *PCD* released a collection entitled *Public Health and Pharmacy: Collaborative Approaches to Improve Population Health*, featuring 17 peer-reviewed articles that bring together cutting-edge work at the interface of pharmacy and public health (3). The collection documents some of the innovative work being done by pharmacists to improve population health. The articles are grouped first to summarize research approaches and contributions and then to describe gaps in research that need to be filled to strengthen the evidence base for the unique role of pharmacy in improving population health. The collection is a call to researchers and professionals in pharmacy and public health to evaluate and publish their work in hopes of expanding on what is already known and being done.

And finally, over the past 5 years, *PCD* has received an increase in submissions addressing aspects of mental health and chronic disease. On December 10 we released the collection, *Mental Health is a Global Public Health Issue* (4). This collection features peer-reviewed articles that examine the relationship between mental health and a variety of factors: family history, self-care practices, sleep, obesity, educational attainment, depression, and COVID-19. Topics featured in this collection represent areas in which we encourage future submissions.

These 4 collections published in 2020 represent our commitment to keep our readership up to date on the latest chronic disease research and prevention findings. We have more exciting collections planned for 2021, on topics such as oral health, global perspectives on improving health and well-being in diverse settings, and health disparities. If you have not already subscribed to *PCD*, we encourage you to do so (https://www.cdc.gov/pcd/subscriptions/index.htm). You will receive the weekly online peer-reviewed journal via email plus monthly email updates of *PCD* articles, the latest calls for papers, announcements of *PCD* collections, and other information.

Finally, we would like to thank our readers, peer reviewers, and growing teams of public health experts who have supported the journal this year and contributed so much to its success. We look forward to 2021 and will continue to identify and publish timely content, improve the quality and reach of *PCD*, and draw in the best expertise to publish statistically and scientifically accurate research that can assist with moving the field of public health forward.
